# Polycystic Ovary Syndrome Among Female Adolescents With Congenital Adrenal Hyperplasia

**DOI:** 10.7759/cureus.20698

**Published:** 2021-12-25

**Authors:** Marwa H Abdelhamed, Waad M Al-Ghamdi, Abdulmoein E Al-Agha

**Affiliations:** 1 Pediatrics, Ain Shams University, Cairo, EGY; 2 Pediatrics, King Abdulaziz University, Jeddah, SAU

**Keywords:** children, adrenocorticotropic hormone, pelvic ultrasonography, androgens, congenital adrenal hyperplasia, polycystic ovary syndrome

## Abstract

Objectives: Polycystic ovary syndrome is a common endocrine disease in adolescent females that is usually diagnosed based on clinical and hormonal abnormalities. Female adolescents with poorly controlled congenital adrenal hyperplasia are at increased risk of developing polycystic ovary syndrome. This study aimed to determine the prevalence of polycystic ovary syndrome and assess its relationship with hormonal control among adolescents with congenital adrenal hyperplasia.

Methods: This retrospective descriptive study included 40 pubertal female adolescents aged between 12 and 20 years with at least two years after menarche diagnosed with classical congenital adrenal hyperplasia since birth who were screened routinely for polycystic ovary syndrome via pelvic ultrasonography between 2012 and 2020 at King Abdul-Aziz University Hospital, Jeddah, Saudi Arabia. Serum adrenocorticotropic hormone, 17-hydroxy -progesterone, testosterone, dehydroepiandrosterone sulfate, luteinizing hormone, and follicle-stimulating hormone levels were measured.

Results: Polycystic ovary syndrome was detected via routine pelvic ultrasonography in 12/40 (30%) females. The median age of the affected females was 16.6 years, with the youngest female aged 12.5 years. The bone age of the patients had advanced ≤3 years. Further, serum adrenocorticotropic hormone was determined to be an independent factor affecting polycystic ovary syndrome development, indicating poor hormonal control (P = 0.005).

Conclusion: Polycystic ovary disease is likely a complication of poorly controlled congenital adrenal hyperplasia disease. Therefore, increasing the awareness of polycystic ovary disease among congenital adrenal hyperplasia females via routine ultrasonography screening is advisable to facilitate the early diagnosis and improve disease management.

## Introduction

Congenital adrenal hyperplasia (CAH) is an autosomal recessive disorder caused by a deficiency in one of the adrenal enzymes of the steroidogenesis pathway. The consequences of CAH have decreased aldosterone and cortisol levels, and hyper-production of adrenal androgens. The incidence of this disease ranges from 1:5000 to 1:15,000 [1]. Polycystic ovary syndrome (PCOS) is a common endocrine and metabolic disorder that is influenced by both genetic and environmental factors [2]. The diagnosis of PCOS should be based on the presence of at least two of the subsequent three criteria: chronic anovulation, hyperandrogenism (clinical or biochemical), or polycystic ovaries [3].

In patients with CAH, abnormal adrenal steroidogenesis affects the development of PCOS. These patients have a reduced synthesis of cortisol. As a result, adrenocorticotropic hormone (ACTH) secretion increases, which promotes adrenal gland stimulation and leads to increased androgen secretion [4]. Due to virilizing consequences of excessive androgen production, affected girls may display hirsutism, male-pattern alopecia, and menstrual abnormalities [5]. Further, obesity plays a major role in disease pathogenesis [6]. Few studies assessing PCOS in female adolescents with CAH have been conducted; however, the prevalence of PCOS in such patients remains unclear. This study aimed to determine the prevalence of PCOS and assess its association with hormonal control and medication adherence among female adolescents with CAH.

## Materials and methods

Patients and clinical characteristics

This retrospective descriptive study included 40 post-pubertal female patients with CAH, who were regularly followed up at the Pediatric Endocrinology outpatient clinic of King Abdul-Aziz University Hospital (KAUH), Jeddah, Saudi Arabia, between 2012 and 2020. The Exclusion criteria were as follows: Pre-pubertal females with CAH, those who did not meet PCOS diagnostic criteria, patients lost throughout routine follow-up, or those with inadequate data in their electronic medical records. All females with CAH were diagnosed based on clinical and laboratory manifestations. Clinical examinations of all study participants were performed by an experienced endocrinologist.

Anthropometric measurements

Height was measured in the standing position, without shoes, using a stadiometer (sensitivity, 0.1 cm). Weight was measured using a portable scale (sensitivity, 0.1 kg) with the patient dressed in light clothing. Body mass index (BMI) was calculated using the Quetelet's index [7], as follows: body weight (kg)/height (m^2^). For children aged 5-19 years, BMI z-scores were assigned based on age and sex to determine whether individuals were overweight (≥1 standard deviation [SD]) and obese (≥2 SD) [8,9].

Tanner staging

Pubertal assessments using Tanner staging were performed on all study participants with CAH to evaluate the presence of external sexual characteristics in the following five stages [10]: Stage one, no pubic hair, no palpable glandular breast tissue. Stage 2, downy pubic hair, palpable breast bud under the areola (an initial sign of puberty in girls). Stage 3, scant, sparse pubic hair, breast elevation with possible papillae occurring as small mounds, no areolar development. Stage 4, terminal dark, coarse pubic hair filling the triangular area that overlies the pubis, areola elevated above the contour of the breast forming a double-scoop appearance. Stage 5, terminal adult type and quantity hair extending into the medial aspect of the thigh, the areolar mound recedes into a single breast contour with hyperpigmentation of the areola, continued papillae development, and nipple protrusion. Puberty normally begins in girls aged 8-13 years. The mean age of thelarche and pubarche among normal girls is 8-10 years and 12 years, respectively, while the mean age of menarche is 13 years [11].

Hyperandrogenism

Hyperandrogenism in patients with PCOS was identified based on biochemical and clinical abnormalities including acne, menstrual irregularity, hirsutism, scalp baldness, and seborrhea [12].

Androgen's normal values including both testosterone level and DHEA-S level in females based on local lab reference at KAUH are shown in Table 1.

**Table 1:** Androgens normal values in females based on local lab reference at KAUH DHEA-S - dehydroepiandrosterone sulfate, KAUH - King Abdul-Aziz University Hospital

Age	Testosterone level (nmol/L)	DHEA-S level (µmol/L)
8-10.9 years	≤1.2135	≤2.4969
11-17.9 years	≤1.3869	1.0042-8.332
≥18 years	0.0693-1.5602	3.92-10.66

Pelvic ultrasound screening

All patients with CAH underwent routine pelvic ultrasonography, which was performed by a pediatric radiologist using a linear transducer L12-5 (Philips EPIQ, 7G ultrasound machine with Doppler). Sonography was performed to detect the presence of ovarian cysts or follicles by measuring the diameter (cm) of the cyst at its widest dimension. PCOS was defined by the presence of ≥10-12 follicles with a diameter of 2-10 mm arranged either peripherally around or scattered throughout an increased amount of stroma [13] or an ovarian volume of ≥10 cm^3^ in a single ovary [14].

Bone age assessment

Bone age was assessed using a radiograph of the non-dominant hand and wrist for patients with CAH and PCOS, and subsequently correlated with age using the Greulich and Pyle atlas [15].

PCOS diagnosis

Female patients with CAH were diagnosed with PCOS based on the recommendations of the Endocrine Society, including the Rotterdam criteria, which require that two of the following three findings are present: hyperandrogenism, ovulatory dysfunction, or polycystic ovaries [16].

Hormone assay

Data from all patients with PCOS were retrospectively collected. Hormonal assays were conducted every three to six months during the follow-up period. The levels of 17-hydroxyprogesterone (17-OHP) were determined via enzyme immunoassay, and ACTH levels were analyzed via electrochemiluminescence assay (Cobas e411 analyzer, Roche Diagnostics, Germany). Testosterone, dehydroepiandrosterone sulfate (DHEA-S), and luteinizing hormone (LH) levels were measured using chemiluminescence immunoassay (IMMULITE 2000, Siemens Healthcare Diagnostics, Erlangen, Germany). Data were obtained from electronic medical records, and hormone levels were interpreted according to the patient's age.

Medications

In this study, all patients with CAH were prescribed hydrocortisone (10-20 mg/m^2^/day divided into three doses) and fludrocortisone (0.05-0.2 mg/day). A routine follow-up of patients with CAH was carried out every three months. The follow-up included clinical and laboratory evaluations and was accompanied by hydrocortisone dose adjustment as needed. 

Ethical considerations

Ethical approval for the study was obtained from the Institutional Review Board of KAUH prior to its implementation no 643-18. Informed consent was obtained from all study participants (either patients or their parents). Patient confidentiality was maintained in accordance with the Declaration of Helsinki.

Statistical analysis

Data analysis was performed using Statistical Package for Social Sciences (SPSS), version 20.0 (SPSS Inc., Chicago, IL, United States of America). A two-tailed P-value ≤ 0.05 was considered statistically significant. Normally distributed, continuous data were expressed as mean and SD values or median and interquartile range. Data distributions were assessed using the Shapiro-Wilk test. Qualitative variables were presented as frequencies and percentages.

The Student’s t-test and Mann-Whitney test were used to compare continuous variables of the two study groups. Correlation analysis using Spearman's method was used to evaluate the strength of the association between the two quantitative variables. Independent factors affecting the development of PCOS were assessed via multiple logistic regression analyses. A literature review with articles identified using Google Scholar and PubMed databases (search terms: PCOS, congenital adrenal hyperplasia, androgens, pelvic ultrasonography, and ACTH) was performed.

The sample size was calculated using PASS program version 15, setting the two-sided confidence interval (CI) at 95%, and the margin of error at 5%. Result from previous study done by Stikkelbroeck et al. [17] that showed 15% of CAH cases had PCO. Calculation according to these values produced a sample size of 40 cases.

Alzanbagi et al. calculated their study size by determining the number of female patients with CAH in the same center using an Epi-Tools epidemiological calculator, which had a 95% CI [18]. The calculated power of the study was 80%.

## Results

**Figure 1:**
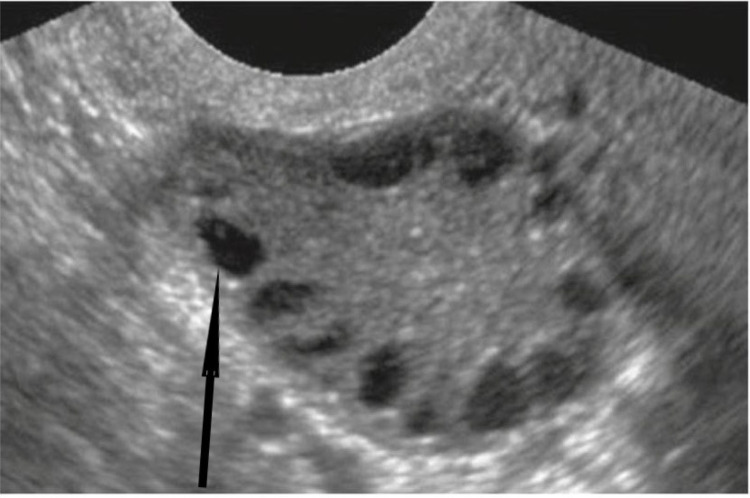
Pelvic ultrasound shows multiple ovarian follicles (arrows) A 14-year-old girl with congenital adrenal hyperplasia and poor hormonal control developed polycystic ovary syndrome (PCOS). Fourteen ovarian follicles fulfilling the PCOS diagnostic criteria are visible via pelvic ultrasound.

Forty female patients with CAH were screened for PCOS using routine pelvic ultrasonography. PCOS was detected in 12/40 patients (30%) during either the initial screening process or follow-up. The mean age of the females diagnosed with PCOS ranged from 12.5 to 20.3 years (mean: 16.56 ± 2.44 years ( Figure 1). Compared to the chronological age of patients, the bone age had advanced ≤ 3 years (>2 SD) in PCOS cases.

Anthropometric measurements

Weight (kg) and height (cm) values of patients in the PCOS and non-PCOS groups varied significantly. The mean weight and height of the PCOS patients was 65.44 ± 21.69 kg (P = 0.004) and 147.83 ± 11.54 cm (P = 0.016), respectively. The BMI of patients with PCOS ranged from 19 to 50 kg/m^2^ (median: 26.2 kg/m^2^). The mean BMI value of females with CAH and PCOS (29.86 ± 9.44 kg/m^2^) was greater than that of female adolescent patients without PCOS (25.56 ± 7.51 kg/m^2^). In addition, the BMI Z-scores of patients with PCOS (1.19 ± 0.96 kg/m^2^) were greater than those of other patients (0.73 ± 1.34 kg/m^2^). Other anthropometric data are displayed in Table 2.

**Table 2:** Clinical characteristics and corresponding hydrocortisone dose of CAH female Patients with PCOS BMI - body mass index, CAH - congenital adrenal hyperplasia, PCOS - polycystic ovary syndrome

#	Age of detection	BMI	BMI Z-score	Tanner stage	Bone age advancement (years)	Hydrocortisone dose in mg/m^2^/day
1	20	27	1.18	V	3.0	17.9
2	20	50	2.39	V	3.0	15.5
3	18.6	22	0.03	V	2.2	10
4	17.6	19	0.90	V	2.3	10.7
5	17.5	29	1.32	V	2.1	18.29
6	17.1	44	2.34	V	2.6	14.15
7	16.1	25	1.09	V	2.0	15.5
8	16.1	24	0.51	V	3.0	20
9	15.1	33	1.79	V	2.1	14
10	14.2	24	1.20	IV	2.0	18.5
11	13.6	37	2.06	IV	2.0	15.6
12	12.5	25	1.24	IV	2.1	20

Tanner staging and hyperandrogenism

Among all CAH female adolescents with detected PCOS, the mean age of thelarche, adrenarche, and menarche were 12.27 ± 1.68, 12.15 ± 1.76, and 13.51 ± 2.33 years, respectively. The mean age of the patients of the PCOS group was greater than those of unaffected girls (P = 0.003). The main characteristics of hyperandrogenism as menstrual irregularities were detected in 90.9% of cases, while hirsutism and acne were found among 60% of cases.

Biochemical profile

**Figure 2:**
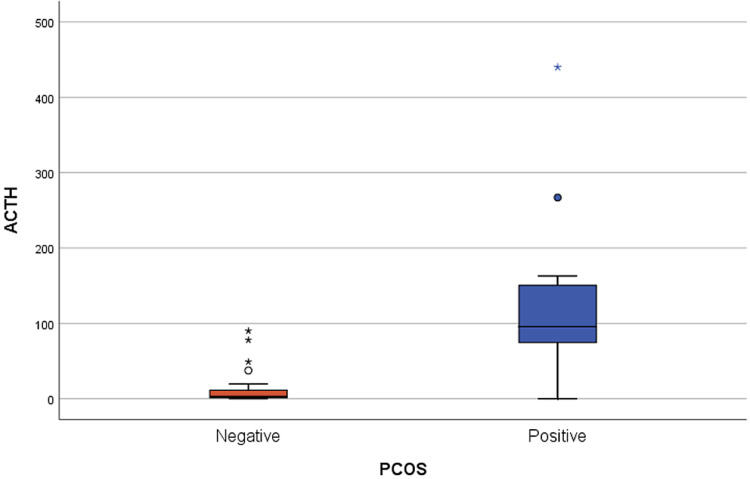
Box-and-whiskers plot of adrenocorticotropic hormone (ACTH) values in patients with polycystic ovary syndrome (PCOS) versus patients without PCOS The box represents the 25th and 75th percentiles, and the whiskers represent the upper and lower adjacent values.

When comparing serum hormonal levels of patients with CAH with and without PCOS, we found that serum levels of 17-OHP (P = 0.006) significantly differed. Further, serum levels of testosterone and DHEA-S varied significantly (P = 0.0001 and P = 0.001, respectively). Similarly, serum levels of ACTH were significantly elevated in patients with PCOS than in those not diagnosed with PCOS (median: 95.8 [74.5-150.5 pmol/L] and 2.8 [1.6-12.6 pmol/L], respectively; p = 0.0001). All patients with CAH who were diagnosed with PCOS had high ACTH levels before PCOS detection, which most likely occurred due to poor medication adherence. Other measured hormone levels are included in Table 3. After adjusting for hormonal factors, ACTH was identified as an independent factor that affected the development of PCOS in patients with CAH (adjusted odds ratio [AOR]: 1.054; CI: 1.018-1.092; P = 0.003). More statistical data using multiple regression analysis are presented in Table 4 and Figure 2.

**Table 3:** Comparison of hormonal assays in patients with and without polycystic ovary syndrome ACTH: adrenocorticotropic hormone, †DHEA-S: dehydroepiandrosterone sulfate, ‡17-OHP: 17-hydroxyprogesterone, §LH: luteinizing hormone, **FSH: follicle-stimulating hormone, ††PCOS: polycystic ovary syndrome, ‡‡SD: standard deviation, §§IQR: interquartile range, ***IU: International Unit.

	PCO										P**	Sig
	Absent					Present						
	Mean	±SD	Median	IQR*		Mean	±SD	Median	IQR*			
ACTH	10.97	19.21	2.8	1.6	12.6	131.21	118.64	95.8	74.5	150.5	0.0001**	HS
Testosterone	1.29	2.85	0.1	0.1	1.0	6.07	3.26	7.2	2.7	8.7	0.0001**	HS
DHEAS	.20	.27	0.1	0.0	.3	9.73	12.39	5.9	0.3	12.9	0.001**	HS
17OH progesterone	3.19	5.08	1.2	0.3	2.9	20.78	24.87	13.4	6.8	23.7	0.006**	HS
LH	5.98	12.75	1.6	0.0	4.4	7.02	3.96	5.5	4.1	11.4	0.01**	S
FSH	4.61	5.12	3.0	0.0	7.2	5.51	1.54	6.0	4.4	6.6	0.288**	NS
Estradiol	154.16	227.22	60.1	0.0	235.0	289.31	182.44	270.4	204.0	336.0	0.014**	S

 

**Table 4:** Multiple regression analysis to study independent factors affecting the development of polycystic ovary syndrome *AOR: adjusted odds ratio, †CI: confidence interval, ‡ACTH: adrenocorticotropic hormone, §DHEA-S: dehydroepiandrosterone sulfate, **17-OHP: 17-hydroxyprogesterone

	Adjusted odds ratio	P	Sig	95% C.I* for AOR	
				Lower	Upper
ACTH	1.054	0.003	HS	1.018	1.092
Testosterone	1.086	0.665	NS	0.747	1.578
DHEAS	1.232	0.580	NS	0.588	2.584
17OH progesterone	1.066	0.568	NS	0.857	1.326

## Discussion

CAH comprises a group of autosomal recessive disorders involving deficiency in enzymes responsible for the synthesis of cortisol, aldosterone, or both, and the accumulation of excessive concentrations of precursor hormones and adrenal androgens [19].

PCOS is one of the most common endocrine disorders affecting females of reproductive age and is characterized by hyperandrogenism, menstrual abnormalities, elevated LH levels, and ovarian cysts [20]. In the current study, PCOS was detected in 12 of the 40 post-pubertal patients. The age of the participants ranged from 12.5 to 20.3 years. The prevalence of PCOS in this study population was 30%; however, Stikkelbroeck et al. [17] reported that polycystic ovaries were detected in two of the 13 patients (15.4%), with ages ranging between 14.8 and 23.5 years. This is different from the prevalence reported by Ghizzoni et al. in 1996 [21], who identified polycystic ovaries in one of the 10 patients with CAH aged 14-24 years. Differences in the ages of patients studied may be a partial explanation for the differences in prevalence reported. Effects of age were explored by Hague et al. [22] who showed that polycystic ovaries were found in four out of 10 (40%) post-pubertal girls with CAH (aged 11-16 years).

Female patients with CAH are at risk of developing PCOS, which affects enzyme activity, and leads to cortisol deficiency. Accordingly, increasing levels of ACTH stimulate the adrenal cortex, as excess accumulation of cortisol precursors increases the synthesis of androgens [23]. Hyperandrogenemia is crucial for disease pathogenesis, which includes morphological changes of the ovaries and clinical syndromes. All processes that result in elevated ovarian androgen levels will lead to the arrest of follicular maturation, follicular atresia, and elevated levels of interstitial tissue in the stroma of the ovaries [24]. Hyperandrogenemia is generally considered to be secondary to poor CAH control, with subsequent chronic elevation of ACTH. The current study confirmed that ACTH is an independent factor that affects the development of PCOS, which validates the hypothesis that poor hormonal control and inadequate suppression of ACTH secretion are etiological factors in the development of PCOS [25]. All serum levels of 17-OHP, and DHEA-S testosterone were higher in patients with PCOS; this indicated adrenal cause as a result of poor hormonal control.

Adolescent patients often do not sufficiently comply with instructions that involve chronic medication use and often miss doses of medications. Furthermore, during puberty, the half-life of hydrocortisone decreases by 50% due to high insulin-like growth factor 1 levels, which diminishes 11β-Hydroxysteroid dehydrogenase activity, and increases cortisol clearance due to glomerular filtration rate enhancement [26]. Another factor that contributes to the development of PCOS is obesity [27]. In the present study, it was noted that patients with PCOS had greater WT and BMI than patients without PCOS. This is consistent with the prior findings reported by Beydoun et al. [28] and Bagheri et al. [29]. In addition, Panidis et al. found that 40%-70% of women with PCOS are either overweight or obese, and the incidence of PCOS in overweight or obese women is four times that of women of normal weight [30].

In this study, girls with CAH who also had PCOS had a variety of clinical manifestations, including irregular menstrual cycles (> 90% of cases) and excess levels of androgens, which manifested as hirsutism, acne, and alopecia. These data are similar to the findings of a study conducted by Hague et al. [22] who assessed post-pubertal girls with CAH. The authors showed that out of the four girls with PCOS, three had irregular menstruation. Menstrual irregularity and secondary amenorrhea with or without hirsutism occur in postmenopausal women, especially those with poor hormonal control [31]. In addition, the prevalence of menstrual abnormalities (93.3%, p < 0.0001) was determined to be higher in a group of patients with PCOS than in patients with CAH [32]. Accelerated bone maturation as a result of hyperandrogenism due to poor hormonal control was demonstrated, and similar findings have been reported previously [31,33-35].

Study limitations are in the form of Inability to fulfill the missing data of some ineligible patients with CAH who have permanently traveled abroad. Some female patients had a lack of clinical manifestations of hyperandrogenism for cosmetic reasons.

## Conclusions

Poor hormonal control in female patients with CAH contributes to the development of PCOS. Increased awareness of the importance of good compliance with medication guidelines is strongly advisable. Routine screening via pelvic ultrasound is recommended for early detection of complications, and to aid the management of the disease. In the upcoming research work, It is also recommended to do a follow-up of patients in the near future after adjustment of treatment.
